# Observational Study on Knowledge and Eating Habits with Respect to Low- and High-FODMAP Foods in Medical Checkup Populations in Japan

**DOI:** 10.3390/nu11102436

**Published:** 2019-10-13

**Authors:** Hiroshi Kaneko, Hirohito Tsuboi, Sayuri Yamamoto, Toshihiro Konagaya

**Affiliations:** 1 Division of Internal Medicine, Hoshigaoka Maternity Hospital, Nagoya 464-0026, Japan; 2 Institute of Medical, Pharmaceutical and Health Science, Kanazawa University, Kanazawa 920-1192, Japan; 3 Department of Gastroenterology, Aichi Medical University School of Medicine, Nagakute 480-1195, Japan; 4 Department of Internal Medicine, Marine Clinic, Nagoya 460-0002, Japan

**Keywords:** fermentable oligosaccharides, disaccharides, monosaccharides, and polyols (FODMAP), knowledge, lower gastrointestinal problems, medical checkup, Japan

## Abstract

Background: There has been an increasing interest in low fermentable oligosaccharides, disaccharides, monosaccharides, and polyols (FODMAP) diets for irritable bowel syndrome. The aims of the present study were (1) to survey knowledge and eating habits with respect to low- and high-FODMAP foods in a general population, and (2) to analyze the relationship between lower gastrointestinal problems (LGIP, pain and irregular stool habits) and habits/foods for prevention. Methods: The present study was carried out with medical examinees (*n* = 1024) at a clinic in Japan using self-administrated questionnaires. The subjects were asked whether they knew each FODMAP food, eating habit, and cooking habit. Chi-squared tests were used for analyzing the characteristics of LGIP-related factors. Logistic regression models were utilized to select foods which predict LGIP. Results: Women cooking by themselves and who were younger indicated less LGIP (Chai-square for trend). More than 60% reported that they often ate rice, coffee, eggs, pork, chicken, breads, noodles, and onions, which are solely classified as high-FODMAP foods. LGIP was related to age and self-cooking habits in women. Soybeans had the least significant relationship with LGIP (odds ratio (OR) 0.48, 95% confidence interval (CI) 0.32–0.73), followed by seaweed, shellfish, wheat, cow’s milk, green beans, and cauliflower. In contrast, artificial sweeteners were most significantly related with LGIP (OR 1.54, 95% CI 1.16–2.03), followed by margarine and tomato paste. Conclusions: The associations between FODMAP foods and LGPI appeared to be different from the Western results. Furthermore, some diets related to LGIP are suggested.

## 1. Introduction

Irritable bowel syndrome (IBS) is a common gastrointestinal illness characterized by the presence of abdominal pain and altered bowel habits [1,2]. Dietary triggers are common, with up to nine out of 10 individuals reporting that food generates symptoms [3,4]. A review paper introduced five studies showing that dietary changes can improve the gastrointestinal symptoms of approximately two-thirds of individuals with IBS [5]. Dietary management was identified as the first line of therapy [6]. Recently, in Western countries such as Australia and North America, there has been an increasing interest in low fermentable oligosaccharides, disaccharides, monosaccharides, and polyols (FODMAP) diets that can be effective for patients with IBS [7,8,9]. However, low-FODMAP foods vary across geographical regions. No information is provided with respect to knowledge and eating habits of the FODMAP diet in Japan, which might be quite different from those in Southeast Asia and in Western countries, although a low-FODMAP diet has been incorporated into clinical practice guidelines for IBS [10].

Before starting low-FODMAP diet therapy for IBS patients and non-IBS patients, it is important to understand diets related with lower gastrointestinal problems (LGIP, abdominal pain and altered bowel habits) when consulting in primary care [11]. We investigated the current status of the knowledge and eating habits low- and high-FODMAP foods as a preliminary study to obtain a simple overview the association between foods and IBS-like symptoms in a Japanese population. Although Fukudo et al. have published some studies concerning IBS in Japanese populations, the association between foods and IBS symptoms is still uncertain. The aims of the present study are (1) to survey knowledge and eating habits with respect to low- and high-FODMAP foods in medical health check-up populations in Japan in order to determine the background of IBS-like symptoms, and (2) to analyze the relationship between LGIP and foods from the viewpoint of FODMAP for prevention.

## 2. Participants and Methods

### 2.1. Participants

The present study was performed with medical examinees at a clinic in Nagoya, Japan between 2 and 19 December, 2015. Office staff of the clinic asked the 1110 examinees to participate in the current study. The staff handed the self-administered questionnaire to the examinees who consented to participate in the study. The participants completed the questionnaire while they were waiting for their medical checkup. All participants were literate and understood the Japanese language well. The enrollment rate was 91.4% (*n* = 1024). This research was approved by the ethical committee of Hoshigaoka Maternity Hospital in Nagoya, Japan.

### 2.2. Questionnaire

The questionnaire was printed on both sides of A4-sized paper (297 mm × 210 mm) for ease of answering. The questionnaire collected data on sex, age bracket, whether one cooked for oneself, LGIP, and food intake. Cooking habits were evaluated by the question “Do you cook for yourself?” and the answer options available were “Yes, always”, “Yes, almost all the time”, “Sometimes”, or “No”. LGIP was evaluated by the question, “Are you suffering from abdominal pain and disordered defecation (constipation or diarrhea)?” The answer options were the same as those for cooking habits. Name, address, ID, and date of test that could identify the individual were not obtained.

Food selection was made by referring to the list of low and high FODMAP foods (186 foods) released from Stanford Hospital & Clinics [12], in which 73 foods written in the Monash University protocol were included [13]. Twenty-six foods were excluded due to the difficulty in obtaining them in Japan. The translation of diets from English to Japanese was performed by a national registered dietitian. Nine foods (including crustaceans, snap beans, melons) were excluded by a counter-translation process completed by a researcher who did not know the original list in English. By searching Japanese websites related to health and medical care on the Internet [14], six foods were added; consequently, 154 low- and high-FODMAP foods were recruited in the present study. The subjects were asked to rank the eating habits in the following four categories for all of the diets; “Know and eat often”, “Know and eat sometimes”, “Know but do not eat”, and “Do not know.” To evaluate the concordance of answers, “Blueberries” was written in the “Meat, non-dairy alternatives” as well as the “Fruits” categories.” Incidentally, the article on the FODMAP diet in East and Southeast Asia was published soon after the present research [15]. Among six foods from Japanese web sites on FODMAP, three were deleted and two were corrected from high- to low-FODMAP.

### 2.3. Statistical Analysis

Submitted questionnaire sheets without sex, or age-bracket were excluded for analysis. The Japanese version of the IBM SPSS Statistics 24 (IBM Japan, Ltd., Tokyo, Japan) and SAS 9.3 (SAS Institute Japan Ltd., Tokyo, Japan) were used to analyze data. A chi-squared test was used to analyze the associations between LGIP and age brackets, and between LGIP and cooking habits. To identify variables that predict LGIP, multiple logistic regression models were applied, where the LGIP (always, almost all the time, sometimes vs. no) was used as a dependent variable and the independent variables were age strata (reference: ages 20–29), sex, and whether one cooked one’s own meals (“Always”, “Almost all the time” vs. “Sometimes”, “No”). Various multiplicity issues which can arise in data analysis were not corrected, with the exception of independent variables in regression models. A *p*-value < 0.05 was considered to indicate statistical significance.

## 3. Results

### 3.1. Demographic Profile

Ten participants out of 1024 were excluded due to them not providing all of the answers in the questionnaire prior to analysis. Nineteen questionnaire sheets were also excluded because they lacked sex, or age-bracket. Consequently, 995 were analyzed. The compliance rate was 97.2%. The characteristics of participants are demonstrated in Table 1. The ratio of gender was almost even (487 males and 508 females). Age distribution was similar between men and women, and peak ages were between 40 and 49. Women more often cooked for themselves in comparison with men (chi-squared = 376.3, *p* < 0.05). Women suffered from LGIP significantly more frequently than men (chi-squared = 52.8, *p* < 0.05).

### 3.2. Recognition of FODMAP Diet and Eating Habit

The supplementary table (Table S1) displays the awareness rate of foods and the frequency of respective food consumption. The number of foods out of 154 which were recognized by participants at a rate less than 50%, 60%, 70%, 80%, and 90% were 16, 21, 28, 36, and 48, respectively. The top ten foods that the participants knew of were rice, spinach, cooking oils, leeks, noodles, tofu, apples, cabbage, eggs, and fish. More than 60% of the subjects often ate rice, coffee, eggs, pork, chicken, breads, noodles, and onions. The kappa value of concordance of “Blueberries” was 0.72.

### 3.3. Factors Related to Gastrointestinal Problem

**Figure 1:**
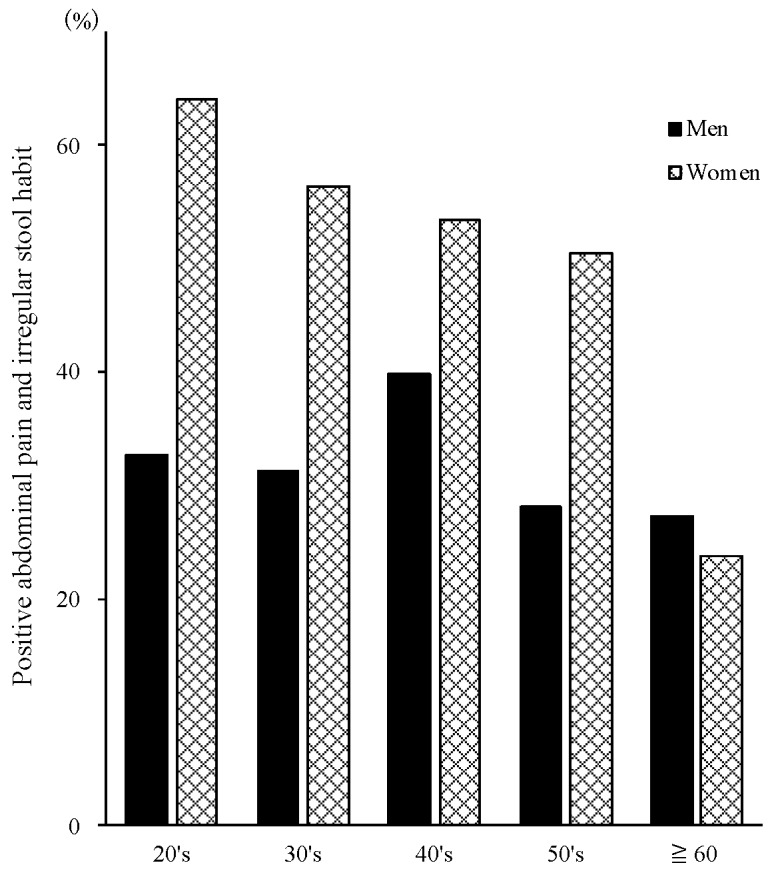
A positive LGIP was more prevalent in the younger generation in women, whereas there were no differences in terms of generations in men. LGIP: lower gastrointestinal problems.

**Figure 2:**
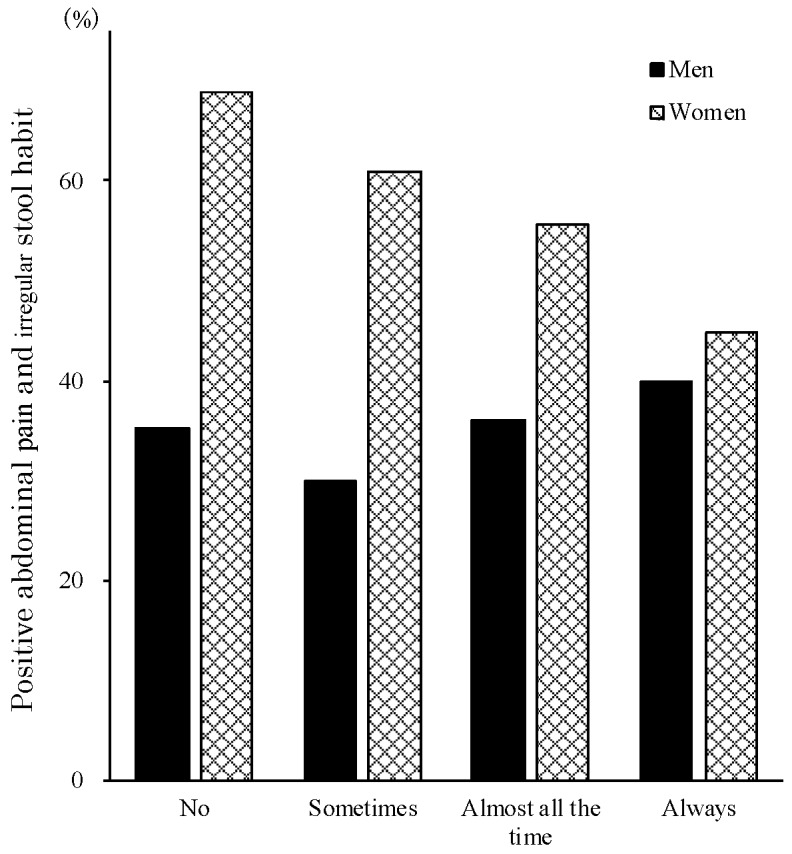
A positive LGIP was more prevalent in women who cooked less, whereas there were no differences in terms of cooking habits in men.

A positive LGIP was more prevalent in the younger generation in women (chi-squared test for trend: *p* < 0.05), whereas there were no differences in terms of generations in men (Figure 1). Simultaneously, a higher frequency of self-cooking habit corresponded to a lower LGIP in women (chi-squared test for trend: *p* < 0.05), whereas there were no differences in terms of cooking habits in men (Figure 2).

### 3.4. Foods That Were Related with Lower and Higher Gastrointestinal Problems

Prior to the analysis to identify foods that are related to LGIP, 48 foods with a degree of participant recognition less than 90% were excluded (Supplementary Materials). The independent variables to predict LGIP significantly are shown in Table 2. The odds ratio (OR) and 95% confidence interval (CI) of each food predicting LGIP after adjusting for covariates (age, sex, whether one cooked one’s own meals) was calculated: soybeans were the least related with LGIP (OR 0.48, 95% CI 0.32–0.73), followed by seaweed (nori) (0.50, 0.25–0.97), shellfish (0.62, 0.43–0.89), wheat (0.63, 0.40–1.00), cow’s milk (0.68, 0.46–0.99), green beans (0.71, 0.51–0.98), and cauliflower (0.75, 0.57–0.98). On the contrary, artificial sweeteners were highly related with LGIP (1.54, 1.16–2.03), followed by margarine (1.40, 1.04–1.87), and tomato paste (1.37, 1.03–1.82).

## 4. Discussion

We investigated the knowledge and eating habits of low- and high-FODMAP foods in medical health check-up populations in Japan. We found that a positive LGIP was more prevalent in the younger generation of women and women without a habit of self-cooking (Figure 1, Figure 2). Further, some high-FODMAP foods detected in the Western studies were associated with less severe abdominal pain and irregular stool habits among the present populations (Table 2). Especially, the most informative finding is that soybeans, which are classified as a high-FODMAP diet in the Western studies, were the most related with a lower LGIP. In relation to the knowledge of FODMAP foods detected in Western countries (high: 63 and low: 91), less than 90% of the present participants did not know of 48 items (26 high-FODMAP and 22 low-FODMAP). The unawareness rate was significantly higher in high-FODMAP (41.2%) compared to low-FODMAP (24.2%) foods (chi-squared = 5.1, *p* < 0.05). This is the first report on the awareness of low and high FODMAP foods in the Japanese population in medical checkups. The high enrollment rate of 91.4% (1024 out of 1110 examinees), high compliance rate of 97.2% (995 valid response out of 1024 participants), and the high kappa vale of concordance of “blueberries” (0.72) suggested that the present results were highly reliable. As written in the method section, we added “Blueberries” into “Meat, non-dairy alternatives, fruits category” as a dummy in addition to the “Fruits” category to check the reliability of answers to the questionnaire.

Women were most likely to report confidence with foods, techniques, or dishes [16]. One Internet survey on self-cooking in Japanese populations (between 20 to 69 years old) conducted in 2011 demonstrated that “Always or almost all the time”, “Sometimes”, and “No” values in men versus women were 17.5% vs. 81.8%, 35.1% vs. 12.3%, and 47.7% vs. 5.9%, respectively [17]. A significantly higher rate of self-catering in women compared to men in the present study was in accordance with the previous data. In the present study, women suffered from LGIP significantly more frequently than men. The finding is compatible with the review that the best-documented risk factor for IBS is female sex, which is associated with an odds ratio of 1.67 (95% CI: 1.53–1.82) across many population-based studies [18].

The FODMAP diet varies markedly across geographical regions. The current database of food content specifically addressing FODMAPs is predominantly from Australia, North America, the United Kingdom, and Scandinavian countries [7,8,9]. Even though the traditional dietary patterns of many Asian cultures are likely to change along with rapid economic growth in Asia and the introduction of Western culture and food consumption patterns, this raises obvious questions about the applicability of the diet to Japanese cuisine and the FODMAP content of food eaten in those regions [15]. Recent meta-analyses and systematic reviews, which indicated a good effect of a low FODMAP diet on IBS patients [7], prompted an increasing need to modify the diet for practical use in other countries [19]. In addition, a low-FODMAP diet has been incorporated into clinical practice guidelines for IBS in Japan [10]. Gibson et al. pointed out that in East and Southeast Asia, health professionals may lack the knowledge to deliver such therapy in practice, although educational resources are already available and can be translated to suit Asian communities [15]. However, to our best knowledge, no highly scientific information on a low FODMAP diet, except for a recently published book on low FODMAP foods written in Japanese by Uno [20], is available in Japan. Therefore, in the present study, we investigated the awareness of high- and low-FODMAP foods in Japan. As a result, one-third of FODMAP foods listed in Western countries were not known of in Japan. More than 60% of the subjects reported that they often ate rice, coffee, eggs, pork, chicken, breads, noodles, and onions. Gibson et al. cited that noodles and gyoza (dumplings) are common in Japanese cuisine aside from fish and sushi, and that the former two foods are generally made using dough of wheat flour—a high-FODMAP ingredient [10]. In addition, bread is a high-FODMAP food. However, in the low- and high-FODMAP foods (186 foods) released from Stanford Hospital & Clinics, noodles and breads are classified as low-FODMAP foods. Moreover, different names are used for similar products in different countries. Cooking methods might modulate FODMAP ingredients, and a small amount of high FODMAP content may create a few problems [10]. Therefore, detailed information on FODMAP content is needed in FODMAP-based dietary intervention in Japan.

In order to analyze the relationship between low- and high FODMAP foods and IBS in future research, the independent variables to predict LGIP, which are among the characteristic signs and symptoms of IBS, were investigated in this study. Soybeans, seaweed (nori), shellfish, wheat, cow’s milk, green beans, and cauliflower were less related with LGIP (Table 2). In contrast, artificial sweeteners, margarine, and tomato paste were more related with LGIP. It has been reported that polyols, which are sugar alcohols found in certain fruits, vegetables, and artificial sweeteners, can induce dose-dependent symptoms of flatulence, abdominal discomfort, and laxative effects when consumed by both healthy volunteers and patients with IBS [21]. It seems reasonable that artificial sweeteners were highly related with LGIP in the present study. In contrast, in this study, LGIP was significantly less related with soybeans and wheat, which are classified as high-FODMAP foods and are generally eaten often in Japan. The putative and possible hypothesis is that people with LGIP might avoid eating them to prevent uncomfortable lower abdominal symptoms.

The limitations of the present study are as follows: first, the questionnaire did not include the current drug usage, past history of abdominal surgery, and pain due to menstruation, which might affect the LGIP. Second, the relationship between a certain diet checked in the current study and LGIP does not indicate a causal relationship due to the cross-sectional study design. Third, this study only enrolled a health check-up population, prohibiting the generalization of the results.

## 5. Conclusions

In spite of these limitations, the novel findings in terms of awareness and eating habits from the viewpoints of high- and low-FODMAP foods should provide some clues to clarify whether dietary therapy for low-FODMAP foods in Japan will be useful for IBS patients in the future. In summary, two-thirds of FODMAP foods were recognized by more than 90% of participants. LGIP was related to age and self-cooking habits in women. The relationship between a certain diet and LGIP was different from Western studies for some foods. Further investigation is warranted to clarify the causal relationship in Japan.

## Figures and Tables

**Table 1:** Characteristics of the analyzed population (*n* = 995).

		Men	Women
		487 (48.9%)	508 (51.1%)
**Age (*n* = 995)**	20–29	89 (18.3%)	79 (15.6%)
	30–39	113 (23.2%)	135 (26.6%)
	40–49	144 (29.6%)	160 (31.5%)
	50–59	96 (19.7%)	113 (22.2%)
	60–69	41 (8.4%)	18 (3.5%)
	70–70 above	4 (0.8%)	3 (0.6%)
**Cook by oneself ^(a)^ (*n* = 990)**	Always	10 (2.1%)	196 (28.7%)
	Almost all the time	37 (7.6%)	144 (28.5%)
	Sometimes	215 (44.2%)	121 (23.9%)
	No	224 (46.1%)	45 (8.9%)
Suffering from LGIP * ^(b)^ (*n* = 984)	Always	9 (1.9%)	33 (6.6%)
	Almost all the time	13 (2.7%)	40 (8.0%)
	Sometimes	139 (28.6%)	197 (39.3%)
	No	324 (66.8%)	231 (46.1%)

* LGIP: lower gastrointestinal problems (abdominal pain and irregular stool habits). ^(a)^ Women more often cooked for themselves in comparison with men. (chi-squared = 376.3, *p* < 0.05). ^(b)^ Women more suffered from LGIP in comparison with men. (chi-squared = 52.8, *p* < 0.05).

**Table 2:** Diets that reduce or aggravate abdominal pain and irregular stool habit.

Diet	Odds Ratio	95% Confidence Interval	*p*-Value
Soybeans *	0.48	0.32–0.73	<0.001
Seaweed (Nori)	0.50	0.25–0.97	0.04
Shellfish	0.62	0.43–0.89	0.01
Wheat *	0.63	0.40–1.00	0.04
Cow’s milk *	0.68	0.46–0.99	0.04
Green beans	0.71	0.51–0.98	0.03
Cauliflower *	0.75	0.57–0.98	0.03
Artificial sweeteners *	1.54	1.16–2.03	0.002
Margarine	1.39	1.04–1.87	0.02
Tomato paste *	1.37	1.03–1.82	0.03

Each result was controlling for age, sex, and cooking habits. * Classified as a high fermentable oligo saccharides, disaccharides, monosaccharides, and polyols (FODMAP) diet.

## Supplementary Materials

The following are available online at https://www.mdpi.com/2072-6643/11/10/2436/s1, Table S1: Awareness rate of food and the frequency of each food consumption.
