# Supplementary material for: Observational Study on Knowledge and Eating Habits with Respect to Low- and High-FODMAP Foods in Medical Checkup Populations in Japan
Source: Nutrients. 2019 Oct 13;11(10):2436. doi: 10.3390/nu11102436 (PMC6835958; doi:10.3390/nu11102436)
Supplement: Supplementary file 1 [file nutrients-11-02436-s001.pdf]

Supplement

Table S1. Awareness rate of food and the frequency of each food consumption.

Category	Diet		*n*	Know (*n* (%))			Do not know (*n* (%))	
				Eat often	Eat sometimes	Do not eat		
Eggs, Meats, Poultry, Fish	Fish		1011	514 (50.8)	479 (47.4)	16 (1.6)	2 (0.2)	
	Eggs		1010	717 (71.0)	280 (27.7)	11 (1.1)	2 (0.2)	
	Beef		1011	451 (44.6)	523 (51.7)	33 (3.3)	4 (0.4)	
	Chicken		1008	702 (69.6)	278 (27.6)	24 (2.4)	4 (0.4)	
	Pork		1009	715 (70.9)	275 (27.3)	14 (1.4)	5 (0.5)	
	Shellfish		1002	140 (14.0)	705 (70.4)	148 (14.8)	9 (0.9)	
	Lamb		1006	4 (0.4)	183 (18.2)	762 (75.7)	57 (5.7)	
	Turkey		1000	0 (0.0)	71 (7.1)	848 (84.8)	81 (8.1)	
	High-fructose corn syrup	*	991	57 (5.8)	201 (20.3)	268 (27.0)	465 (46.9)	#
Dairy	Chocolate	*	1011	369 (36.5)	545 (53.9)	89 (8.8)	8 (0.8)	
	Ice cream	*	1006	196 (19.5)	693 (68.9)	108 (10.7)	9 (0.9)	
	Cow's milk	*	1002	417 (41.6)	437 (43.6)	138 (13.8)	10 (1.0)	
	Custard	*	1003	73 (7.3)	699 (69.7)	210 (20.9)	21 (2.1)	
	Creamy sauces	*	992	48 (4.8)	576 (58.1)	289 (29.1)	79 (8.0)	
	Whipped cream		1006	55 (5.5)	605 (60.1)	319 (31.7)	27 (2.7)	
	Sherbet		1004	34 (3.4)	596 (59.4)	345 (34.4)	29 (2.9)	
	Mozzarella cheese		1006	86 (8.5)	634 (63.0)	251 (25.0)	35 (3.5)	
	Cream cheese		1002	49 (4.9)	574 (57.3)	337 (33.6)	42 (4.2)	
	Cheddar cheese		1006	65 (6.5)	511 (50.8)	341 (33.9)	89 (8.8)	
	Sour cream	*	1004	10 (1.0)	285 (28.4)	610 (60.8)	99 (9.9)	
	Buttermilk	*	1000	16 (1.6)	236 (23.6)	553 (55.3)	195 (19.5)	#
	Ricotta cheese	*	1001	8 (0.8)	255 (25.5)	468 (46.8)	270 (27.0)	#
	Goat’s milk	*	994	0 (0.0)	14 (1.4)	661 (66.5)	319 (32.1)	#
Meat, Non-dairy alternatives	Tofu		1012	599 (59.2)	392 (38.7)	18 (1.8)	3 (0.3)	
	Miso	*	1007	610 (60.6)	378 (37.5)	14 (1.4)	5 (0.5)	
	Peanut		1009	85 (8.4)	726 (72.0)	188 (18.6)	10 (1.0)	
	Soybeans	*	1011	314 (31.1)	575 (56.9)	111 (11.0)	11 (1.1)	
	Macadamia nuts		1008	49 (4.9)	642 (63.7)	296 (29.4)	21 (2.1)	
	Broad bean	*	1007	18 (1.8)	593 (58.9)	374 (37.1)	22 (2.2)	

	Soy milk	*	1001	133 (13.3)	422 (42.2)	422 (42.2)	24 (2.4)	
	Cashews	*	1006	50 (5.0)	566 (56.3)	339 (33.7)	51 (5.1)	
	Pistachios	*	1008	25 (2.5)	513 (50.9)	414 (41.1)	56 (5.6)	
	Walnut		1005	23 (2.3)	265 (26.4)	353 (35.1)	364 (36.2)	#
	Tempeh		1002	3 (0.3)	35 (3.5)	254 (25.3)	710 (70.9)	#
	Kidney bean	*	994	2 (0.2)	35 (3.5)	149 (15.0)	808 (81.3)	#
**Grains**	Noodles		1011	649 (64.2)	350 (34.6)	9 (0.9)	3 (0.3)	
	Breads		1009	657 (65.1)	327 (32.4)	21 (2.1)	4 (0.4)	
	Rice		1011	851 (84.2)	150 (14.8)	6 (0.6)	4 (0.4)	
	Pastas		1008	478 (47.4)	489 (48.5)	32 (3.2)	9 (0.9)	
	Chips		1004	154 (15.3)	616 (61.4)	221 (22.0)	13 (1.3)	
	Wheat	*	1010	372 (36.8)	530 (52.5)	92 (9.1)	16 (1.6)	
	Pancakes		1006	41 (4.1)	609 (60.5)	338 (33.6)	18 (1.8)	
	Biscuits		1007	78 (7.7)	652 (64.7)	258 (25.6)	19 (1.9)	
	Waffles		1007	22 (2.2)	561 (55.7)	397 (39.4)	27 (2.7)	
	Crackers		1006	25 (2.5)	531 (52.8)	421 (41.8)	29 (2.9)	
	Popcorn		1007	21 (2.1)	518 (51.4)	438 (43.5)	30 (3.0)	
	Barley	*	1007	39 (3.9)	475 (47.2)	453 (45.0)	40 (4.0)	
	Rye	*	1005	19 (1.9)	353 (35.1)	559 (55.6)	74 (7.4)	
	Cereals		1010	89 (8.8)	426 (42.2)	400 (39.6)	95 (9.4)	
	Bagels		1008	37 (3.7)	427 (42.4)	437 (43.4)	107 (10.6)	#
	Oatmeal		1007	7 (0.7)	151 (15.0)	649 (64.4)	200 (19.9)	#
	Tortillas		996	5 (0.5)	242 (24.3)	479 (48.1)	270 (27.1)	#
	Pretzels		1005	6 (0.6)	298 (29.7)	389 (38.7)	312 (31.0)	#
	Chicory root	*	1004	1 (0.1)	81 (8.1)	367 (36.6)	555 (55.3)	#
	Quinoa		1002	7 (0.7)	50 (5.0)	307 (30.6)	638 (63.7)	#
	Inulin	*	1006	0 (0.0)	9 (0.9)	98 (9.7)	899 (89.4)	#
**Fruits**	Apples	*	1004	342 (34.1)	575 (57.3)	85 (8.5)	2 (0.2)	
	Bananas		1002	331 (33.0)	537 (53.6)	129 (12.9)	5 (0.5)	
	Orange		1001	135 (13.5)	704 (70.3)	154 (15.4)	8 (0.8)	
	Strawberries		1001	167 (16.7)	687 (68.6)	137 (13.7)	10 (1.0)	
	Peaches	*	1001	71 (7.1)	700 (69.9)	219 (21.9)	11 (1.1)	
	Grapes		1002	95 (9.5)	706 (70.5)	190 (19.0)	11 (1.1)	
	Watermelon	*	1002	97 (9.7)	619 (61.8)	274 (27.3)	12 (1.2)	
	Kiwi		1002	131 (13.1)	614 (61.3)	245 (24.5)	12 (1.2)	
	Nashi pears	*	1001	131 (13.1)	699 (69.8)	157 (15.7)	14 (1.4)	
	Persimmon	*	1001	136 (13.6)	550 (54.9)	301 (30.1)	14 (1.4)	
	Pineapple		999	79 (7.9)	698 (69.9)	208 (20.8)	14 (1.4)	
	Blueberries		998	98 (9.8)	594 (59.5)	292 (29.3)	14 (1.4)	

	Mango	*	997	28 (2.8)	534 (53.6)	415 (41.6)	20 (2.0)	
	Lemon		997	46 (4.6)	569 (57.1)	362 (36.3)	20 (2.0)	
	Figs	*	999	29 (2.9)	356 (35.6)	584 (58.5)	30 (3.0)	
	Canned fruit	*	1000	21 (2.1)	391 (39.1)	557 (55.7)	31 (3.1)	
	Papaya	*	996	6 (0.6)	243 (24.4)	713 (71.6)	34 (3.4)	
	Pears	*	999	24 (2.4)	407 (40.7)	534 (53.5)	34 (3.4)	
	Dried fruits	*	999	61 (6.1)	446 (44.6)	453 (45.3)	39 (3.9)	
	Lychee		996	14 (1.4)	395 (39.7)	542 (54.4)	45 (4.5)	
	Prunes	*	999	39 (3.9)	347 (34.7)	563 (56.4)	50 (5.0)	
	Raspberries		997	9 (0.9)	353 (35.4)	581 (58.3)	54 (5.4)	
	Lime		996	4 (0.4)	235 (23.6)	697 (70.0)	60 (6.0)	
	Tangerine		997	16 (1.6)	345 (34.6)	543 (54.5)	93 (9.3)	
	Plums	*	998	13 (1.3)	302 (30.3)	585 (58.6)	98 (9.8)	
	Passion fruit		998	4 (0.4)	188 (18.8)	688 (68.9)	118 (11.8)	#
	Guava	*	998	4 (0.4)	116 (11.6)	732 (73.3)	146 (14.6)	#
	Cranberries		998	11 (1.1)	282 (28.3)	556 (55.7)	149 (14.9)	#
	Applesauce	*	997	12 (1.2)	230 (23.1)	592 (59.4)	163 (16.3)	#
	Apricots	*	999	4 (0.4)	199 (19.9)	593 (59.4)	203 (20.3)	#
	Blackberries	*	1000	3 (0.3)	172 (17.2)	580 (58.0)	245 (24.5)	#
	Dates	*	997	1 (0.1)	59 (5.9)	543 (54.5)	394 (39.5)	#
	Mandarin		997	9 (0.9)	90 (9.0)	455 (45.6)	443 (44.4)	#
	Nectarines	*	996	3 (0.3)	113 (11.3)	415 (41.7)	465 (46.7)	#
	Honeydew		995	13 (1.3)	194 (19.5)	295 (29.6)	493 (49.5)	#
	Rhubarb		996	1 (0.1)	34 (3.4)	186 (18.7)	775 (77.8)	#
	Boysenberries	*	998	1 (0.1)	16 (1.6)	172 (17.2)	809 (81.1)	#
	Cantaloupe		997	4 (0.4)	14 (1.4)	85 (8.5)	894 (89.7)	#
**Vegetables**	Cabbage		1001	580 (57.9)	401 (40.1)	18 (1.8)	2 (0.2)	
	Spinach		1001	385 (38.5)	563 (56.2)	49 (4.9)	4 (0.4)	
	Onion	*	1002	601 (60.0)	368 (36.7)	28 (2.8)	5 (0.5)	
	Eggplant		1002	317 (31.6)	596 (59.5)	84 (8.4)	5 (0.5)	
	Tomatoes		1002	528 (52.7)	406 (40.5)	63 (6.3)	5 (0.5)	
	Carrots		1002	492 (49.1)	461 (46.0)	43 (4.3)	6 (0.6)	
	Bell peppers		1000	301 (30.1)	618 (61.8)	74 (7.4)	7 (0.7)	
	Seaweed (nori)		1001	347 (34.7)	608 (60.7)	39 (3.9)	7 (0.7)	
	Cucumbers		1000	437 (43.7)	491 (49.1)	65 (6.5)	7 (0.7)	
	Lettuce		1002	482 (48.1)	478 (47.7)	35 (3.5)	7 (0.7)	
	Bamboo shoots		999	78 (7.8)	740 (74.1)	173 (17.3)	8 (0.8)	
	Broccoli		1001	411 (41.1)	489 (48.9)	92 (9.2)	9 (0.9)	
	Turnips		997	79 (7.9)	625 (62.7)	276 (27.7)	17 (1.7)	
	Bok choy		999	121 (12.1)	696 (69.7)	162 (16.2)	20 (2.0)	

	Mushrooms	*	996	63 (6.3)	633 (63.6)	279 (28.0)	21 (2.1)	
	Green beans		998	108 (10.8)	648 (64.9)	221 (22.1)	21 (2.1)	
	Cauliflower	*	997	55 (5.5)	483 (48.4)	432 (43.3)	27 (2.7)	
	Zucchini		997	46 (4.6)	372 (37.3)	510 (51.2)	69 (6.9)	
	Sugar snap peas	*	995	81 (8.1)	487 (48.9)	280 (28.1)	147 (14.8)	#
	Kale		995	7 (0.7)	122 (12.3)	546 (54.9)	320 (32.2)	#
	Bean sprouts		994	29 (2.9)	214 (21.5)	306 (30.8)	445 (44.8)	#
	Alfalfa		995	11 (1.1)	144 (14.5)	296 (29.7)	544 (54.7)	#
	Artichokes	*	995	1 (0.1)	65 (6.5)	340 (34.2)	589 (59.2)	#
	Water chestnuts		996	1 (0.1)	28 (2.8)	321 (32.2)	646 (64.9)	#
**Beverages**	Coffee		1001	711 (71.0)	220 (22.0)	64 (6.4)	6 (0.6)	
	Tea		997	271 (27.2)	531 (53.3)	185 (18.6)	10 (1.0)	
	Port wine	*	991	15 (1.5)	145 (14.6)	555 (56.0)	276 (27.9)	#
	Sherry wine	*	988	1 (0.1)	52 (5.3)	655 (66.3)	280 (28.3)	#
**Seasonings, Condiments**	Leeks	*	998	385 (38.6)	551 (55.2)	59 (5.9)	3 (0.3)	
	Butter		1002	235 (23.5)	650 (64.9)	112 (11.2)	5 (0.5)	
	Salt		1003	584 (58.2)	403 (40.2)	11 (1.1)	5 (0.5)	
	Mayonnaise		1003	339 (33.8)	583 (58.1)	75 (7.5)	6 (0.6)	
	Onion (green part)		1002	506 (50.5)	439 (43.8)	51 (5.1)	6 (0.6)	
	Pepper		1003	494 (49.3)	465 (46.4)	37 (3.7)	7 (0.7)	
	Garlic	*	998	274 (27.5)	591 (59.2)	125 (12.5)	8 (0.8)	
	Honey	*	997	131 (13.1)	601 (60.3)	255 (25.6)	10 (1.0)	
	Sesame		1002	359 (35.8)	565 (56.4)	67 (6.7)	11 (1.1)	
	Mustard		1000	88 (8.8)	689 (68.9)	205 (20.5)	18 (1.8)	
	Margarine		1002	175 (17.5)	522 (52.1)	286 (28.5)	19 (1.9)	
	Olives		997	173 (17.4)	515 (51.7)	289 (29.0)	20 (2.0)	
	Cooking oils		1000	345 (34.5)	539 (53.9)	86 (8.6)	30 (3.0)	
	Herbs		998	80 (8.0)	514 (51.5)	352 (35.3)	52 (5.2)	
	Garlic powders	*	998	65 (6.5)	422 (42.3)	447 (44.8)	64 (6.4)	
	Tomato paste	*	997	86 (8.6)	496 (49.7)	348 (34.9)	67 (6.7)	
	Artificial sweeteners	*	998	72 (7.2)	404 (40.5)	431 (43.2)	91 (9.1)	
	Pumpkin seeds		997	5 (0.5)	184 (18.5)	712 (71.4)	96 (9.6)	
	Garlic/onion infused oil		999	108 (10.8)	433 (43.3)	361 (36.1)	97 (9.7)	
	Xylitol	*	999	44 (4.4)	386 (38.6)	457 (45.7)	112 (11.2)	#
	Sunflower seeds		993	1 (0.1)	107 (10.8)	747 (75.2)	138 (13.9)	#
	Garlic salt	*	994	41 (4.1)	340 (34.2)	466 (46.9)	147 (14.8)	#
	Onion powders	*	995	11 (1.1)	188 (18.9)	609 (61.2)	187 (18.8)	#
	Onion salt	*	994	6 (0.6)	185 (18.6)	574 (57.7)	229 (23.0)	#

Chives		996	42 (4.2)	350 (35.1)	325 (32.6)	279 (28.0)	#
Molasses	*	997	18 (1.8)	238 (23.9)	435 (43.6)	306 (30.7)	#
Broth (homemade)		997	7 (0.7)	111 (11.1)	557 (55.9)	322 (32.3)	#
Flax seeds		997	2 (0.2)	44 (4.4)	326 (32.7)	625 (62.7)	#
Sorbitol	*	992	5 (0.5)	62 (6.3)	197 (19.9)	728 (73.4)	#
Chia seeds		995	18 (1.8)	38 (3.8)	202 (20.3)	737 (74.1)	#
Mannitol	*	995	0 (0.0)	12 (1.2)	143 (14.4)	840 (84.4)	#
Isomalt	*	993	0 (0.0)	13 (1.3)	101 (10.2)	879 (88.5)	#

* Classified as high-FODMAP food, # Foods that 10% or less participants know.
